# Heterogeneously-integrated lasers on thin film lithium niobate

**DOI:** 10.1515/nanoph-2025-0458

**Published:** 2025-11-28

**Authors:** Mingxiao Li, Chao Xiang, Joel Guo, Jonathan Peters, Mario Dumont, Shixin Xue, Jeremy Staffa, Qili Hu, Zhengdong Gao, Qiang Lin, John E. Bowers

**Affiliations:** Department of Electrical and Computer Engineering, 8786 University of California Santa Barbara, Santa Barbara, CA 93106, USA; Department of Electrical and Computer Engineering, University of Rochester, Rochester, NY 14627, USA; Institute of Optics, University of Rochester, Rochester, NY 14627, USA

**Keywords:** semiconductor lasers, lithium niobate, integrated photonics

## Abstract

We demonstrate a versatile heterogeneous integration platform unifying III–V gain with thin-film lithium niobate (TFLN) photonic circuits to create high-performance lasers with integrated functionality. This breakthrough overcomes the critical barrier to fully integrated photonic systems by combining optical gain, low-loss cavities, and phase control on a single chip. We present two distinct laser architectures: a distributed feedback laser achieving 11.0 kHz intrinsic linewidth and 4.0 mW in-fiber power through self-injection locking to a high-*Q* TFLN resonator, and a Vernier ring laser exhibiting 44 nm continuous tuning range with > 40 dB side-mode suppression ratio. Crucially, the heterogeneous integration of the gain section with TFLN’s components provides a promising path to implementing direct intracavity modulation, which is a functionality that typically requires discrete components. This inherent capability makes our platform a foundational advancement for future compact, robust systems in coherent communications, ultrafast optical metrology, quantum photonic processors, and microwave photonic systems operating at GHz bandwidths, marking a significant advancement toward complete photonic system integration.

## 1 Introduction

Photonic integration is the cornerstone of system miniaturization, a prerequisite for next-generation technologies. This drive has established monolithic electronic-photonic co-integration as the definitive path to achieving radical reductions in size, weight, power consumption, and cost (SWaP-C). Among integration strategies, heterogeneous integration stands out for its ability to unite disparate material systems, each optimized for a specific function. Silicon photonics exemplifies this, leveraging mature semiconductor manufacturing for the high-volume production of optical transceivers, quantum systems, and sensors [1], [2], [3].

The integration of semiconductor lasers has been pivotal in this progress, enabling breakthroughs in optical communications, signal processing, and sensing. The primary architectures for semiconductor lasers are purely III–V-based or involve direct growth on silicon [4], [5]. Recently, significant effort has been devoted to the heterogeneous integration of III–V materials with passive platforms, adopting an external cavity design. This approach decouples the lossy gain region from the passive cavity, significantly enhancing laser performance by extending the optical mode into low-loss materials such as silicon nitride (SiN) [6], [7]. Thin-film lithium niobate (TFLN) has emerged as a particularly compelling platform, offering a unique combination of a broad optical transparency window, a high refractive index, strong second- and third-order nonlinearities for wavelength conversion, and a strong linear Pockels effect for ultrafast electro-optic modulation [8], [9]. Integrating TFLN into photonic systems thus promises unprecedented performance for optical communications, nonlinear optics, and quantum technologies.

Concurrently, advances in wafer-scale processing have enabled semiconductor lasers to dramatically reduce SWaP-C while achieving narrow linewidths that rival state-of-the-art fiber lasers [10], [11], [12], [13]. This degree of coherence is indispensable for precision spectroscopy, optical atomic clocks, quantum information processing, and coherent communications [14], [15], [16], [17], [18], [19]. However, these high-performance lasers often remain standalone components. Their full functionality typically depends on external circuits for critical functions such as frequency stabilization, phase shift, and modulation, which severely throttles system miniaturization and compromises stability [20]. This reliance on discrete components represents a major bottleneck for the development of compact, robust, and fully integrated photonic systems.

These prospects have spurred further development of laser technology. However, despite TFLN’s exceptional electro-optic coefficient and compatibility with heterogeneous integration, the efficient integration of optical gain with fast electro-optic control remains a critical barrier to realizing fully single-chip systems. As a result, researchers have investigated various integration approaches, including hybrid integration, transfer printing, flip-chip bonding, and dielectric adhesive layer bonding [21], [22], [23], [24], [25], [26], [27], [28]. In this work, we overcome this challenge through the heterogeneous integration of III–V semiconductor optical amplifiers with TFLN photonic circuits. We demonstrate integrated distributed feedback (DFB) and Vernier ring lasers on TFLN that unite optical gain and cavity resonance on a single chip, which enables direct electro-optic functionality that previously necessitated bulky, external components.

## 2 Results

### 2.1 Laser design

The heterogeneous III–V/TFLN platform provides a significant advantage: the ability to monolithically define high-performance optical cavities and mirrors directly within the photonic circuit. This eliminates the performance limitations and reliability concerns associated with cleaved facets or hybridly attached external mirrors, while also facilitating the co-integration of monitor photodetectors (PDs) for on-chip feedback and wafer-scale testing.

To capitalize on these benefits, we designed and realized two key laser architectures: a distributed feedback laser for stable single-frequency operation and a Vernier-ring-based laser for wide tunability. Both leverage intimate III–V/TFLN integration to move beyond standalone sources and create lasers with intrinsic, high-performance functionality.

**Figure 1:**
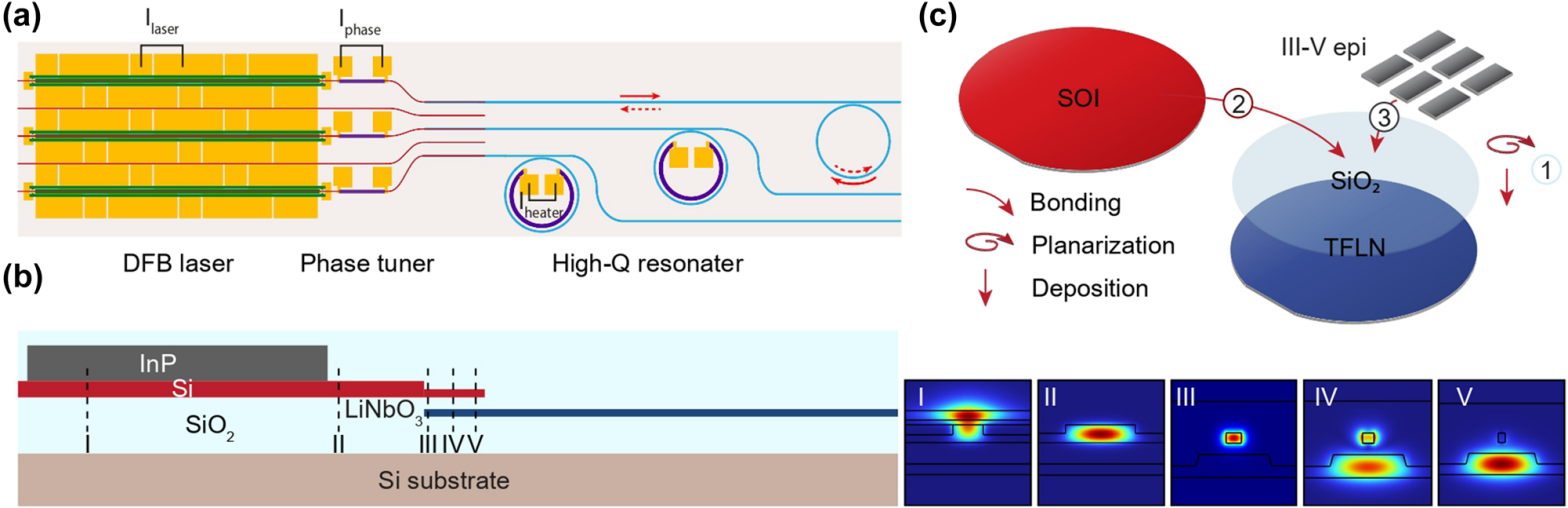
Architecture and fabrication of the heterogeneously-integrated lasers on thin film lithium niobate. (a) Schematic of III–V/TFLN platform with DFB lasers, phase tuners, and high-*Q* microresonators on a common substrate. A continuous-wave signal emitted from the laser is coupled into the microresonator and partially backscattered. The backscattered signal is reinjected into the laser, triggering self-injection locking. The locking is optimized by controlling the laser current and phase tuner current. (b) Simplified device cross section. The laser is based on InP/Si gain section, and the microresonator is based on TFLN. An intermediate Si layer delivers light from the InP/Si layer to the TFLN layer. Right: simulated optical mode distribution in the device at labeled position. (c) A schematic of the fabrication process using 4-inch wafers.

A schematic of the general laser structure is shown in Figure 1(a). Light is transferred from the gain section to the TFLN cavity through a Si-based tapered coupler, optimized to minimize intracavity loss as shown in Figure 1(b). The output waveguides on both sides are angled at 10° to suppress back-reflection and ensure stable lasing.

#### 2.1.1 DFB laser

For applications requiring stable, single-frequency emission, such as coherent communications and sensing, we implemented a DFB laser. The design capitalizes on the excellent lithographic control available in our heterogeneous platform to pattern a high-contrast Bragg grating directly alongside the III–V gain section on the Si waveguide. A narrow-band grating response with high side-lobe suppression is critical, as it directly dictates the laser’s linewidth and side-mode suppression ratio (SMSR). The low propagation loss of the TFLN waveguides is essential to form a high-quality factor (*Q*) cavity, which is necessary for narrow linewidth performance.

In our design, the grating is defined in the Si layer adjacent to the gain section. To achieve high single-mode selectivity and output power, the coupling coefficient *κ* is carefully designed to provide an optimal *κL* product. The measured performance of this device is presented in the following section. The high SMSR and output power achieved make this laser an ideal on-chip pump source for integrated nonlinear optics, leveraging TFLN’s strong *χ* ^(2)^ nonlinearity for applications such as second-harmonic generation or optical parametric oscillations.

For a standard first-order DFB laser (without a phase shift), the key characteristics are derived from its photonic bandgap structure. The lasing modes occur at the edges of this stopband. To force lasing at the Bragg wavelength *λ*_0_ and achieve a single longitudinal mode, a phase shift *π*/2 is introduced into the grating using a quarter-wave shift. This defect in the periodic structure creates a resonant state in the middle of the bandgap [11].

For a first-order DFB laser with a quarter-wave shift, the key characteristics are defined by its threshold gain and photonic bandgap. The mirror loss for the lasing mode at the Bragg wavelength *λ*_0_ is given by:

$$\alpha_{m}L=2\sinh^{-1}\left(\kappa L\right),$$

where *κ* is the coupling coefficient of the grating, *L* is the length of the grating, and *α*_*m*_ is the mirror loss coefficient.

The width of the photonic bandgap (stopband) is given by:

$$\Delta \lambda =\frac{\lambda_{0}^{2}}{\pi n_{g}}\kappa ,$$

where *λ*_0_ is the center wavelength, *n*_*g*_ is the group index and Δ*λ* is the width of the stopband.

Compared to DBR lasers that typically use a *κL* lower than 1, DFB lasers require higher *κL* values to provide sufficient distributed feedback without discrete mirrors. The higher *κL* creates a larger stopband that strongly suppresses side modes, ensuring stable single-mode operation under varying operating conditions. However, there is a trade-off: excessively high *κL* introduces substantial mirror loss (*α*_*m*_ ∝ sinh^−1^(*κL*)), which increases the threshold current and reduces slope efficiency, potentially leading to spatial hole burning and multimoding. In our devices, we selected *κL* = 3 to balance these competing factors.

#### 2.1.2 Vernier ring laser

To address the need for wavelength agility in WDM systems and precision sensing, we designed a widely tunable laser based on the Vernier effect. This architecture fully exploits the heterogeneous integration of low-loss TFLN waveguides to achieve both narrow linewidth and wide tunability.

Silicon was specifically selected for Vernier rings to capitalize on its large thermo-optical coefficient (d*n*/d*T*), which enables efficient low-power thermal tuning of the resonant wavelengths via integrated microheaters [9]. Resistive microheaters are placed in proximity to two Si rings of slightly different radii. By independently controlling their temperatures, the resonant peaks of the two rings can be aligned at a single wavelength, which experiences high transmission through the filter [29]. The large Vernier effect created by the two rings enables a broad tuning range far exceeding that of a single ring. Crucially, the entire cavity, including the gain section, passive TFLN waveguides, and tunable filter, is fabricated monolithically, ensuring stability and compactness. This design unites the wide tunability traditionally associated with external cavity lasers with the miniaturization and robustness of a fully integrated photonic circuit. The performance of this tunable laser is detailed in the results section.

### 2.2 Laser performance

#### 2.2.1 DFB laser

**Figure 2:**
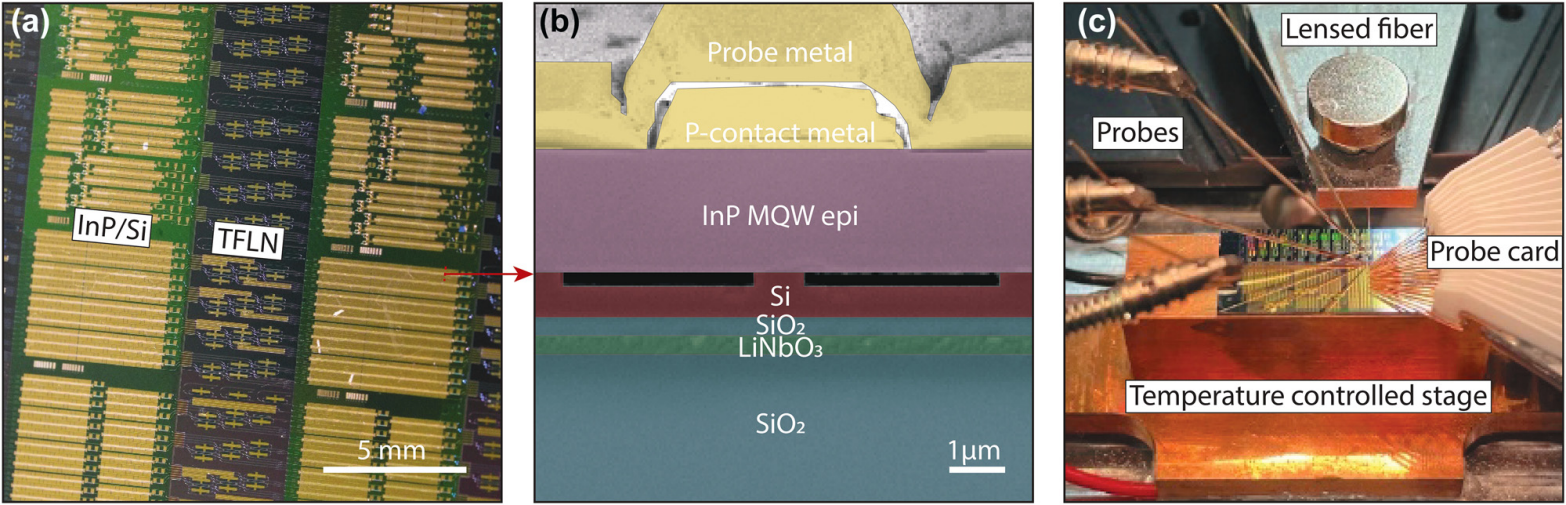
Wafer-scale III–V/TFLN laser array, device structure, and measurement setup. (a) A photo of an array of heterogeneously integrated lasers processed on a 4-inch wafer. Green: bonded single crystalline Si layer. Yellow: gold probe metal and radio-frequency signal electrodes. Dark blue: lithium niobate thin film. (b) False-coloured cross section of the multiple quantum well distributed feedback laser with lithium niobate. Image is taken by SEM. The red arrow highlights the section where the SEM was taken. (c) Measurement setup. A diced chip is placed on a temperature stabilized motion stage; a probe card is adopted for laser current supply; four direct current probes are used for heater and phase shifter control; a motion controlled lensed fiber is employed for output light coupling.

A brief fabrication process is shown in Figure 1(c). The details of the heterogeneous integration process to realize our device are provided in the Methods section. Devices with grating couplers are also placed in the layout to monitor the change in loss related to fabrication during the process [30]. Figure 2(a) shows the devices on the 4-inch wafer after the heterogeneous process. The wafer is diced and polished for testing. The scanning electron microscopy (SEM) image in Figure 2(b) shows the cross section of our heterogeneous structure.

The experiment setup is shown in Figure 2(c). A probe card is first adopted for the laser current supply. A direct current is then applied to the phase shifter using a set of probes for self-injection locked laser control. While another set of probes apply the current for resonance wavelength tuning to match the lasing wavelength.

**Figure 3:**
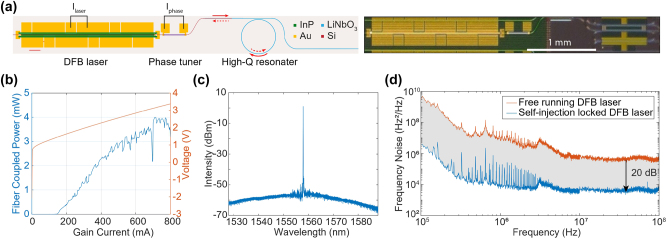
Performance characterization of heterogeneously-integrated III–V/TFLN DFB lasers. (a) Left: schematic of the heterogeneously integrated DFB laser. Right: a photo of the lasers and resonators processed on a 4-inch wafer. (b) Measured LIV curve of the DFB laser on TFLN. (c) The single mode lasing spectrum measured by an optical signal analyzer (OSA). (d) The measured frequency noise power spectral density from an OEwave noise analyzer of the laser.

Figure 3(a) shows the laser structure, which is formed by taper-coupling a III–V semiconductor optical amplifier (SOA) epi to TFLN layer that integrates a phase shifter, a high-*Q* resonator, and an output coupling waveguide. This laser utilizes a hybrid InP/Si active waveguide for efficient optical mode coupling to the underlying silicon layer. The laser output is subsequently transferred to a TFLN layer via a silicon mode transition taper, where a high-*Q* LN resonator reduces the laser noise through self-injection locking. The output power is measured using a lensed fiber that collects light from a tapered waveguide at the LN facet.

The light-current curve (L–I) in Figure 3(b) shows a lasing threshold of 150 mA and a maximum fiber-coupled power of 4.0 mW at a drive current of 700 mA. Accounting for a 6.7 dB chip-to-fiber coupling loss, this corresponds to 18.7 mW of on-chip power [31]. The irregular variations in the L–I curve are attributed to mode hopping, and the distinct dip indicates the point of laser-resonator wavelength matching. The optical spectrum in Figure 3(c) confirms single-mode operation at 1,559.0 nm with an SMSR exceeding 50 dB.

The laser’s spectral purity was characterized by measuring its frequency noise power spectral density. Above 100 kHz, the frequency noise spectrum transitions to a white noise floor (*S*_*ν*_(*f*) ≈ constant) extending to approximately 100 MHz. This fundamental noise limit arises from quantum phase diffusion due to spontaneous emission events, which produce random phase jumps with a white power spectral density. The magnitude of this noise floor determines the laser’s intrinsic linewidth Δ*ν* through the Schawlow-Townes relation:

$$\Delta \nu =\pi S_{\nu }\left(f\right)\;\text{for}\;f\gg f_{c},$$

where *f*_*c*_ is the corner frequency where flicker noise transitions to white noise [32]. In its free-running state, the DFB laser exhibited a white frequency noise floor of 0.33 MHz^2^/Hz at an offset frequency of 40 MHz (Figure 3(d)), equivalent to an intrinsic linewidth of 1.0 MHz. This quantum-limited noise floor represents the ultimate spectral purity limit for the laser oscillator.

To significantly enhance the laser’s coherence, we employed the self-injection locking technique. This was achieved through a two-step process: First, the lasing wavelength was aligned with the resonance of a high-*Q* lithium niobate resonator with a loaded *Q* of 3 × 10^5^; subsequently, the optical phase between the laser and the resonator was precisely optimized using the integrated phase shifter. This locking mechanism dramatically suppressed the laser’s phase noise. The effectiveness of this technique is evident in the noise spectrum, where the stabilized laser shows a drastically reduced noise floor of 3.5 kHz^2^/Hz, a suppression factor of nearly two orders of magnitude, as shown in Figure 3(d). This new noise level yields a remarkably narrow intrinsic linewidth of 11.0 kHz. The current degree of noise reduction is primarily limited by the quality factor of the lithium niobate resonator. Future improvements to the resonator’s design and fabrication are expected to yield even higher *Q* factors and, consequently, further reduction of the laser linewidth. Details of the derivation are provided in the Methods.

#### 2.2.2 Vernier ring laser

**Figure 4:**
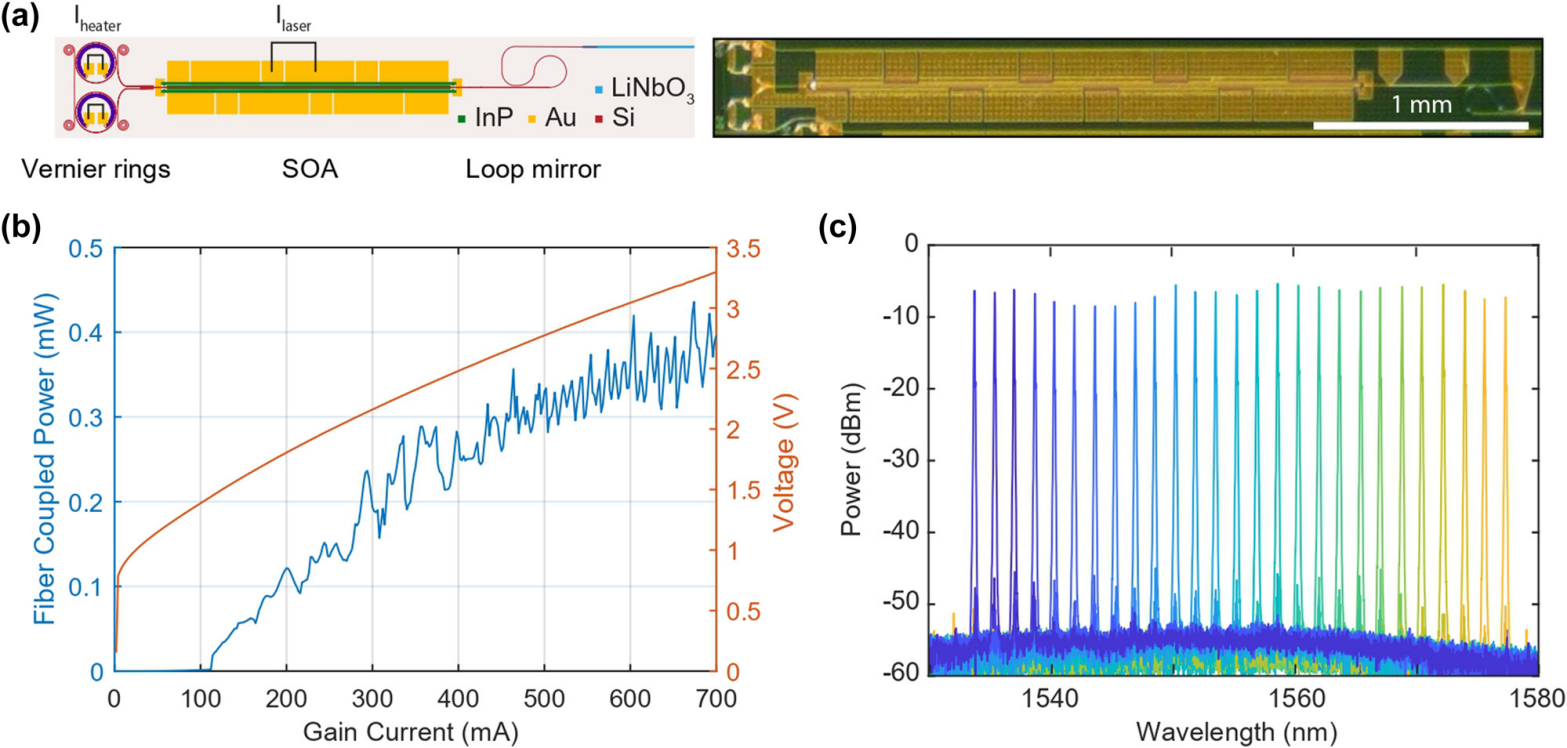
Performance characterization of heterogeneously-integrated III–V/TFLN Vernier ring lasers. (a) Left: schematic of the heterogeneously integrated Vernier ring laser. Right: a photo of the lasers processed on a 4-inch wafer. (b) Measured LIV curve of the Vernier ring laser on TFLN. (c) The course tuning spectra showing the tuning range of the laser measured by an optical signal analyzer (OSA).

In addition to the DFB design, the same heterogeneous platform facilitates a widely tunable laser source based on the Vernier effect. This architecture employs two cascaded, thermally tunable silicon microring resonators with slightly different free spectral ranges (FSRs) of 215 GHz and 208 GHz as shown in Figure 4(a). Tuning is achieved by leveraging the thermo-optic effect in Si ring resonators. The Vernier effect creates a combined transmission spectrum in which only a single mode, aligned across both rings, experiences minimal loss and reaches the lasing threshold, thereby providing highly selective wavelength filtering.

The laser performance is characterized by a threshold current of 110 mA. At the operating point, it delivers a fiber-coupled output power of 0.43 mW as shown in Figure 4(b). Given the consistent 6.7 dB chip-to-fiber coupling loss inherent to the packaging setup, this translates to an on-chip power of 2.01 mW. The primary advantage of this design is its extensive tunability. As shown in Figure 4(c), by applying current to the microheaters atop each ring to exploit the thermo-optic effect, we achieved coarse wavelength tuning over a remarkable 44-nm range, spanning from 1,533 to 1,577 nm, covering the entire C-band and extending into the L-band. Throughout this tuning range, the Vernier filter mechanism robustly maintains single-mode operation, as evidenced by an SMSR consistently greater than 40 dB.

The free-running linewidth of this tunable laser is measured to be approximately 200 kHz due to the low loaded *Q* of 4 × 10^4^ in the Si rings. It is important to note that this specific Vernier-ring design did not incorporate a lithium niobate resonator for self-injection locking (SIL), which precluded the active noise suppression demonstrated in the DFB design. Consequently, the linewidth performance represents a direct trade-off, sacrificing the ultra-narrow linewidth achievable through SIL for the paramount benefit of exceptionally wide, continuous tuning within a single, compact device.

## 3 Discussion

The results presented demonstrate the versatility of our heterogeneously integrated III–V/TFLN platform in producing two distinct classes of high-performance lasers, each optimized for different application spaces.

The DFB laser architecture, enhanced by self-injection locking to a high-*Q* TFLN resonator, achieves a narrow intrinsic linewidth of 11.0 kHz. Further improvement for exceptional coherence has been demonstrated in other works, which could be adopted in future works [7], [13], [31]. The ultra-low loss properties of the TFLN passive cavity make this platform ideal for applications demanding high spectral purity, such as coherent optical communications, high-resolution spectroscopy, and as a stable local oscillator. The estimated on-chip power of 18.7 mW is sufficient to serve as a pump for integrated nonlinear processes inherent to the TFLN platform, such as second-harmonic generation or optical parametric oscillation.

Conversely, the Vernier ring laser prioritizes wide tunability over ultimate spectral purity. By leveraging the strong thermo-optic effect in silicon, this design achieves a coarse tuning range of 44 nm while maintaining a respectable around 200 kHz free-running linewidth and an SMSR > 40 dB. This performance is attained without the active stabilization used in the DFB design, highlighting the inherent single-mode selectivity of the Vernier filter mechanism. This laser is perfectly suited for applications like wavelength division multiplexing (WDM) systems, optical sensing, and spectroscopy where broad wavelength coverage is paramount.

This work firmly establishes heterogeneous III–V/TFLN integration as a powerful platform to realize fully functional multi-purpose photonic systems on a single chip [33], [34].

## 4 Methods

### 4.1 Fabrication process

**Figure 5:**
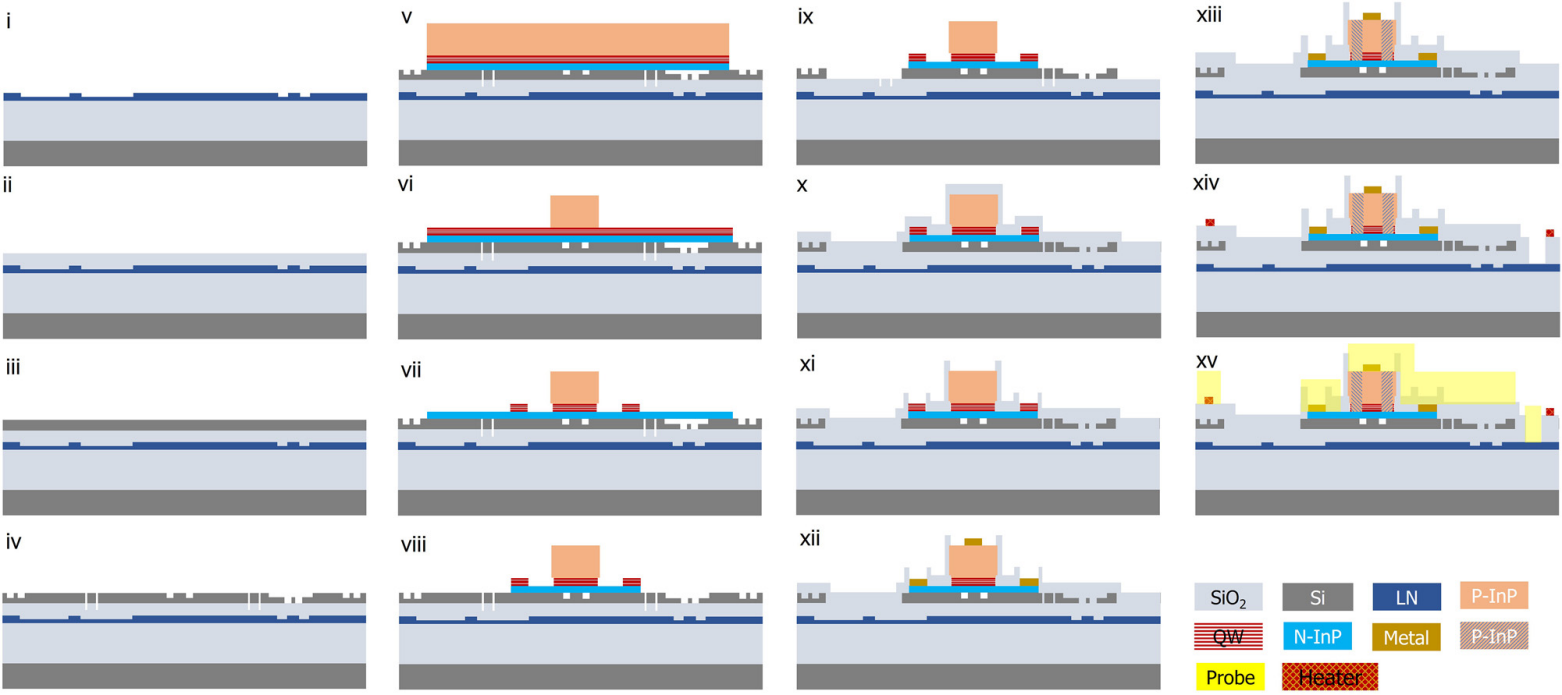
Multilayer heterogeneous integration process. (i) Lithium niobate wafer patterning. (ii) Silicon dioxide cladding buffer layer deposition and planarization with chemical-mechanical polishing (CMP). (iii) SOI wafer bonding and substrate removal. (iv) Defining gratings and mode transition taper on single crystaline silicon. (v) InP MQW epi pieces bonding. (vi) P-InP etch of bonded epi pieces. (vii) Mesa etch. (viii) N-InP etch. (ix) Excess Si removal. (x) Silicon dioxide deposition. (xi) Silicon dioxide etch for Vias opening. (xii) P- and N-metal contacts formation. (xiii) Laser passivation. (xiv) Heaters deposition. (xv) Probe metal formation. All the photolithography steps are performed with a DUV stepper, except for the fine Si grating writing, which uses electron beam lithography. Elements are not shown to scale.

As shown in Figure 5, the entire structure comprises three distinct layers fabricated through two separate bonding processes. The devices were fabricated on thin film *x*-cut single crystalline lithium niobate (600 nm thick, from NanoLN) bonded to a 4.7 μm silicon dioxide layer atop a silicon substrate. Fabrication commenced with waveguide patterning: a resist layer was spin-coated and baked, followed by the photolithographic definition of the waveguide structures. The samples were then subjected to argon ions (Ar^+^) dry etching to form 320-nm-deep ridge waveguides. Subsequent cleaning removed residual resist and redeposited material, yielding smooth waveguide sidewalls. A 600 nm thick SiO_2_ cladding was deposited via ICP-PECVD and planarized using chemical-mechanical polishing (CMP).

Next, a single-crystalline silicon-on-insulator (SOI) stack was directly bonded to the polished SiO_2_ surface on the LN thin film, after which the SOI substrate and buried oxide were removed, forming a Si/LN heterogeneous structure. Following patterning of loop mirrors and optical routing structures on crystalline silicon, an InP-based multiple quantum well (MQW) epitaxial layer was directly bonded to the silicon as the gain medium for the extended distributed Bragg reflector laser. Multiple etch steps-including methane/hydrogen/argon reactive ion etching (MHA) and wet etching-defined the laser architecture (P-InP, MQW, and N-InP regions). Oxide deposition and reactive ion etching (RIE) formed vias for metallization, whereupon multiple metal layers were deposited to supply current to lasers and heaters. Proton implantation passivation established the MQW current channel. Finally, the wafer was diced and facet-polished for characterization.

### 4.2 Derivation of the DFB laser linewidth equation

The fundamental limit of semiconductor laser linewidth is governed by the modified Schawlow-Townes-Henry relation. Following the rate equation approach, the Lorentzian linewidth can be derived from the spontaneous emission contribution to phase noise [35]:

(1) $$\Delta \nu =\frac{R_{sp}}{4\pi n_{p}}\left(1+\alpha_{H}^{2}\right),$$

where *R*_*sp*_ is the spontaneous emission rate into the lasing mode and *n*_*p*_ is the number of photons in the cavity.

The spontaneous emission rate is given by:

(2) $$R_{sp}=v_{g}g_{th}n_{sp},$$

where *v*_*g*_ is the group velocity, *g*_*th*_ is the threshold gain, and *n*_*sp*_ is the spontaneous emission factor.

At threshold, the gain equals the total loss:

(3) $$g_{th}=\alpha_{i}+\alpha_{m},$$

where *α*_*i*_ is the internal loss and *α*_*m*_ is the mirror loss.

The photon number *n*_*p*_ is related to the output power by considering the power balance. The output power from one facet is:

(4) $$P_{\text{out}}=\frac{1}{2}v_{g}\alpha_{m}h\nu n_{p},$$

where the factor of 1/2 accounts for power emitted through one facet (assuming symmetric output).

Substituting these relations into Eq. (1):

(5) $$\Delta \nu =\frac{h\nu v_{g}^{2}n_{sp}}{8\pi P_{\text{out}}}\left(1+\alpha_{H}^{2}\right)\alpha_{m}\left(\alpha_{i}+\alpha_{m}\right).$$

For a distributed feedback (DFB) laser with a first-order, *λ*/4-shifted grating, the mirror loss is [11]:

(6) $$\alpha_{m}=\frac{2}{L}\sinh^{-1}\left(\kappa L\right),$$

The derivation assumes steady-state operation where the output power *P*_out_ is treated as an independent parameter. In practice, for a given pumping level, both the intracavity photon density *n*_*p*_ and output power *P*_out_ depend on the total cavity loss (*α*_*i*_ + *α*_*m*_) and the pumping current. However, Eq. (5) remains valid as it expresses the linewidth for a laser operating at a specified output power level, which is the typical experimental condition.

### 4.3 Self-injection locking to a passive resonator

The derived Schawlow–Townes equation (Eq. (5)) represents the fundamental free-running linewidth of the DFB laser. A powerful technique to surpass this limit and achieve ultra-narrow linewidth is self-injection locking to a high-*Q* external resonator, such as a microring or microdisk.

The self-injection locking phenomenon occurs when light from a laser is back-reflected from a high-*Q* resonator and re-injects into the laser cavity, leading to significant noise reduction and linewidth narrowing. The complete derivation follows the coupled mode theory approach with careful attention to the laser-resonator interaction [31], [36].

#### 4.3.1 Coupled mode equations

The dynamics of the fundamental mode inside a passive optical resonator is described by the following equation for the cavity field amplitude *a*_1_:

(7) $$\frac{da_{1}}{dt}=\left[i\left(\omega_{1}-\omega_{10}\right)-\frac{\kappa_{1\text{t}}}{2}\right]a_{1}+i\sqrt{\kappa_{1\text{e}}}s_{\text{in}},$$

where:

– *ω*_1_ is the laser angular frequency,
– *ω*_10_ is the angular resonance frequency of the fundamental mode,
– *κ*_1t_ = *κ*_1e_ + *κ*_10_ is the total photon decay rate,
– *κ*_1e_ is the external coupling rate,
– *κ*_10_ is the intrinsic loss rate,
– *s*_in_ is the amplitude of the input wave, normalized such that *P*_in_ = *ℏω*_1_|*s*_in_|^2^ represents the input power.

The light inside the resonator can be scattered in a counter-propagating mode, described by the amplitude *b*_1_, with dynamics governed by:

(8) $$\frac{db_{1}}{dt}=\left[i\left(\omega_{1}-\omega_{10}\right)-\frac{\kappa_{1\text{t}}}{2}\right]b_{1}+i\gamma_{\text{bs}}\frac{\kappa_{1\text{t}}}{2}a_{1},$$

where *γ*_bs_ is the normalized backscattering coefficient (|*γ*_bs_| ≪ 1).

#### 4.3.2 Steady-state solution

At steady state (d/d*t* = 0), we solve Eq. (7) for the intracavity field *a*_1_:

(9) $$0=\left[i\left(\omega_{1}-\omega_{10}\right)-\frac{\kappa_{1\text{t}}}{2}\right]a_{1}+i\sqrt{\kappa_{1\text{e}}}s_{\text{in}}.$$

Rearranging terms:

(10) $$a_{1}=\frac{i\sqrt{\kappa_{1\text{e}}}}{-i\left(\omega_{1}-\omega_{10}\right)+\frac{\kappa_{1\text{t}}}{2}}s_{\text{in}}=\frac{2i\sqrt{\kappa_{1\text{e}}}}{\kappa_{1\text{t}}\left(1-i\xi_{1}\right)}s_{\text{in}},$$

where *ξ*_1_ ≡ 2(*ω*_1_ − *ω*_10_)/*κ*_1t_ is the normalized detuning.

Similarly, from Eq. (8), the steady-state backscattered field *b*_1_ is:

(11) $$b_{1}=\frac{i\gamma_{\text{bs}}}{1-i\xi_{1}}a_{1}.$$

#### 4.3.3 Laser locking condition

The backscattered field *b*_1_ couples out of the resonator and re-injects into the laser cavity. This feedback modifies the laser’s oscillation condition. The locking relation is derived from the laser rate equations with external feedback:

(12) $$\omega_{1}=\omega_{\text{L}}-\frac{\kappa_{1\text{t}}}{2}K\;\text{Im}\left[{\text{e}}^{\text{i}\phi }\frac{\kappa_{1\text{t}}}{2}\frac{b_{1}}{i\gamma_{\text{bs}}\left(i\sqrt{\kappa_{1\text{e}}}s_{\text{in}}\right)}\right],$$

where:

– *ω*_L_ is the free-running laser frequency,
– *ϕ* is the round-trip feedback phase,
– *K* is the normalized locking strength.

The locking strength *K* is given by:

(13) $$K=4\frac{Q_{1\text{t}}}{Q_{\text{DFB}}}\frac{\kappa_{1\text{e}}}{\kappa_{1\text{t}}}|\gamma_{\text{bs}}|T\sqrt{1+\alpha_{H}^{2}},$$

where:

– *α*_*H*_ is the amplitude-phase coupling factor (Henry factor),
– *T* is the single-trip transmission between laser and resonator,
– *Q*_1t_ = *ω*_1_/*κ*_1t_ is the loaded *Q* of the resonator,
– *Q*_DFB_ = *ω*_1_/Δ*ν*_DFB_ is the *Q*-factor corresponding to the DFB laser linewidth.

#### 4.3.4 Noise reduction factor derivation

The noise reduction factor $F_{\text{NR}}\equiv S_{\text{DFB}}^{\left(f\right)}/S_{\text{SIL}}^{\left(f\right)}=|\partial \omega_{\text{L}}/\partial \omega_{1}{|}^{2}$ quantifies the phase noise suppression. To derive this, we substitute Eqs. (10) and (11) into the locking condition:

(14) $$\frac{b_{1}}{i\gamma_{\text{bs}}\left(i\sqrt{\kappa_{1\text{e}}}s_{\text{in}}\right)}=\frac{1}{i\sqrt{\kappa_{1\text{e}}}s_{\text{in}}}\cdot \frac{1}{1-i\xi_{1}}a_{1}$$

(15) $$=\frac{2}{\kappa_{1\text{t}}}\cdot \frac{1}{{\left(1-i\xi_{1}\right)}^{2}}.$$

The locking condition simplifies to:

(16) $$\omega_{1}=\omega_{\text{L}}-\frac{\kappa_{1\text{t}}}{2}K\;\text{Im}\left[{\text{e}}^{\text{i}\phi }\frac{1}{{\left(1-i\xi_{1}\right)}^{2}}\right].$$

Let $F\left(\xi_{1}\right)=\text{Im}\left[{\text{e}}^{\text{i}\phi }\frac{1}{{\left(1-i\xi_{1}\right)}^{2}}\right]$ . Then:

(17) $$\omega_{\text{L}}=\omega_{1}+\frac{\kappa_{1\text{t}}}{2}KF\left(\xi_{1}\right).$$

Differentiating with respect to *ω*_1_:

(18) $$\frac{\partial \omega_{\text{L}}}{\partial \omega_{1}}=1+\frac{\kappa_{1\text{t}}}{2}K\frac{\partial F}{\partial \xi_{1}}\frac{\partial \xi_{1}}{\partial \omega_{1}}.$$

Since *ξ*_1_ = 2(*ω*_1_ − *ω*_10_)/*κ*_1t_, we have ∂*ξ*_1_/∂*ω*_1_ = 2/*κ*_1t_, so:

(19) $$\frac{\partial \omega_{\text{L}}}{\partial \omega_{1}}=1+K\frac{\partial F}{\partial \xi_{1}}.$$

At resonance (*ξ*_1_ = 0) and with optimal phase (*ϕ* = 0):

(20) $${\left.\frac{\partial F}{\partial \xi_{1}}\right|}_{\xi_{1}=0}=2\cos\left(0\right)=2.$$

Thus:

(21) $$\frac{\partial \omega_{\text{L}}}{\partial \omega_{1}}=1+2K.$$

For strong locking (*K* ≫ 1):

(22) $$\frac{\partial \omega_{\text{L}}}{\partial \omega_{1}}\approx 2K.$$

The noise reduction factor is:

(23) $$F_{\text{NR}}={\left|\frac{\partial \omega_{\text{L}}}{\partial \omega_{1}}\right|}^{2}\approx 4K^{2}.$$

Substituting the expression for *K* from Eq. (13):

(24) $$F_{\text{NR}}\approx 64\frac{Q_{1\text{t}}^{2}}{Q_{\text{DFB}}^{2}}\frac{\kappa_{1\text{e}}^{2}}{\kappa_{1\text{t}}^{2}}|\gamma_{\text{bs}}{|}^{2}T^{2}\left(1+\alpha_{H}^{2}\right).$$

This result shows that strong noise reduction requires:

– High resonator *Q*-factor (*Q*_1t_),
– Strong backscattering (|*γ*_bs_|),
– Low loss between laser and resonator (*T*),
– Proper phase matching (*ϕ* ≈ 0).

This leads to crucial insight that the linewidth reduction achieved through self-injection locking is proportional to the square of the ratio of the cavity *Q*-factor. Since the *Q*-factor of a microresonator (*Q*_1t_) can be orders of magnitude larger than the *Q*-factor of the solitary DFB cavity (*Q*_DFB_), the resulting locked laser linewidth Δ*ν*_locked_ can be dramatically reduced. Additionally, the linewidth enhancement factor *α*_*H*_ plays a significant role in determining the final noise performance, similar to its effect on the fundamental laser linewidth in Eq. (1).
